# Does Hunger Contribute to Socioeconomic Gradients in Behavior?

**DOI:** 10.3389/fpsyg.2017.00358

**Published:** 2017-03-10

**Authors:** Daniel Nettle

**Affiliations:** Centre for Behaviour and Evolution, Institute of Neuroscience, Newcastle UniversityNewcastle, UK

**Keywords:** socioeconomic position, hunger, food insecurity, impulsivity, ADHD, aggression, anxiety, addiction

## Abstract

Recent research has uncovered many examples of socioeconomic gradients in behavior and psychological states. As yet there is no theoretical consensus on the nature of the causal processes that produce these gradients. Here, I present the *hunger hypothesis*, namely the claim that part of the reason that people of lower socioeconomic position behave and feel as they do is that they are relatively often hungry. The hunger hypothesis applies in particular to impulsivity-hyperactivity, irritability-aggression, anxiety, and persistent narcotic use, all of which have been found to show socioeconomic gradients. I review multiple lines of evidence showing that hunger produces strong increases in these outcomes. I also review the literatures on food insufficiency and food insecurity to show that, within affluent societies, the poor experience a substantial burden of hunger, despite obtaining sufficient or excess calories on average. This leads to the distinctive prediction that hunger is an important mediator of the relationships between socioeconomic variables and the behavioral/psychological outcomes. This approach has a number of far-reaching implications, not least that some behavioral and psychological differences between social groups, though persistent under current economic arrangements, are potentially highly reversible with changes to the distribution of financial resources and food.

## Introduction

The last decade has seen an increasing appreciation that, within affluent societies, socioeconomic position (SEP) is an important predictor of variation in behavioral and psychological outcomes (e.g., Griskevicius et al., 2011a,b; Kraus et al., 2012; Haushofer and Fehr, 2014; Hill et al., 2016). Demonstrations of the central importance of SEP may come as no surprise to sociologists, but within psychology, SEP had been relatively neglected as a source of variation. One reason psychologists may not have been drawn to SEP as an explanatory variable is that, from a psychological perspective, SEP is just a proxy. The success of simple socioeconomic variables as statistical predictors suggests that they must roughly capture some set of inputs or experiences that entrains a specific pattern of psychological processes in response. However, there is as yet no theoretical consensus on what these experiences and processes are.

A general strategy for advancing understanding in this area is to identify states that: (1) occur more commonly in people of lower SEP, for reasons that follow directly from their poverty and disadvantage; and (2) can be demonstrated (independently of SEP) to cause psychological and behavioral shifts of the kind that have been statistically associated with lower SEP. This article proposes that hunger is such a state. It presents the hypothesis that a number of the observed behavioral and psychological differences between people of low and high SEP within affluent societies are partly the consequences of people of low SEP being more often hungry. I am using hunger here to refer to the physiological need to eat food, though in many of the studies reviewed below, what is actually measured is the subjective sensation associated with that need. This is a broader definition of hunger than those used in some sources (e.g., Keys et al., 1950). Note also that hunger (an instantaneous state) is not to be equated with, and does not imply, undernutrition (which can only be evaluated over longer time periods).

Some immediate caveats are in order. The hunger hypothesis does not claim that all socioeconomic gradients in behavior or psychological variables are related to hunger, nor that, in those cases where hunger does play a role, hunger is the sole cause. Occupying lower SEP will have a whole range of (potentially separable) physical, emotional and social-cognitive consequences. Thus, explanations for observed socioeconomic gradients no doubt need to be heterogeneous. Hunger would be, even if the hypothesis were supported, a *partial* explanation for *some* of the observed gradients. This is all the more true since SEP itself is a multi-facetted construct, operationalised in a number of distinct ways (e.g., income, education, occupational status, perceived position on a social ladder). In view of this heterogeneity, it is important to delineate the scope of the hunger hypothesis. I am applying it here to four case studies of clusters of outcomes that show social gradients: impulsivity-hyperactivity, irritability-aggression, anxiety and persistent narcotic use. There are other behaviors and states, such as prosociality (Kraus et al., 2012; Korndörfer et al., 2015) and locus of control (Pepper and Nettle, 2017), that also vary by SEP. Whether hunger has any relationship to these differences is beyond the scope of this paper, and not a claim I wish to imply. There are also SEP differences in general intelligence. The interpretation of these is complex (Deary and Johnson, 2010; Marioni et al., 2014), and though it is possible that hunger has some relevance (see Mani et al., 2013), I do not consider this issue here. The hunger hypothesis is also most readily applicable to socioeconomic gradients defined in terms of income, since, as we shall see below, income is a strong determinant of hunger within affluent societies. Social gradients defined in terms of, for example, education, would only be explicable under the hypothesis to the extent that more education improves income or food access.

In what follows, I first review the evidence that the subjects of my four case studies– namely impulsivity-hyperactivity, irritability-aggression, anxiety and persistent narcotic use—all show SEP gradients within affluent societies. There then follows a review of the effects of hunger on behavior, in which I note that these same four clusters of variables appear consistently, from multiple sources of evidence. I then present evidence that hunger is strongly socioeconomically patterned. This leads to a consideration of alternative versions of the hunger hypothesis, and discussion of their predictions, testability and implications.

## Social Gradients in Behavioral and Psychological Outcomes: Four Case Studies

In this section, I briefly discuss four clusters of behaviors and psychological states that have been recurrently identified in the literature as correlated with SEP, and whose correlations with SEP are not yet explained. My decision to focus on these particular clusters is not, of course, independent of my knowledge of the literature on the psychology of hunger. I could therefore be accused of having chosen the cases that suit the argument. This is in one sense true, but the purpose of this article is hypothesis generation rather than hypothesis testing. Thus it is important to bear in mind that what I present here is grounds for entertaining the hunger hypothesis, not evidence that the hunger hypothesis is correct (and it could turn out to be correct for some of the clusters but not the others). We will return to how the hypothesis can be tested in a later section. The four clusters, although in some cases possibly related, are heterogeneous in type. Narcotic use is a behavior, whereas anxiety is an affective state generally measured by self-report. This heterogeneity is not damaging to the present thesis, since the aim is not to equate the four clusters with one another (each no doubt has a distinct set of causes and consequences), but rather show that a parallel argument for the partially mediating role of hunger in the SEP gradient can be made for each of the four case studies.

### Impulsivity-Hyperactivity

Impulsivity is a term with varied meanings (Reynolds et al., 2006), encompassing both measures of temporal discounting (‘waiting’) and of response inhibition (‘stopping’). Whilst there is evidence of stable, personality-like individual differences in proneness to impulsivity (Odum, 2011), contemporary research has established that impulsivity can also vary markedly in relation to context or the subject’s state (Kidd et al., 2013; Lempert and Phelps, 2016). Lower-SEP individuals have been found to be less patient in terms of waiting (Green et al., 1996; Adams and White, 2009) and more disinhibited in terms of stopping (Paál et al., 2015). The chief motivation for studying the SEP-impulsivity association in adults has been the hypothesis that greater impulsivity statistically mediates the relationships between SEP on the one hand and health-damaging behaviors on the other (Adams and White, 2009; Ward et al., 2009).

Impulsivity is not a psychological disorder. However, exhibiting impulsivity (along with inattention and hyperactivity) is a diagnostic criterion for the disorder attention-deficit hyperactivity disorder (ADHD) in children. Thus, the presence of more ADHD diagnoses in children of lower-SEP is a source of evidence for greater impulsivity amongst children from those groups. And indeed, ADHD is a highly social patterned disorder, more common in families of lower SEP. The odds of an ADHD diagnosis are over twice as high for families in the lowest tertile of SEP compared to the highest tertile (Russell et al., 2015b). In one British study using multiple socioeconomic variables, it was the presence of financial difficulties that was the strongest predictor of ADHD diagnosis (Russell et al., 2015a). Given that there is a greater rate of ADHD diagnosis in children from low-SEP groups, it is a reasonable inference that there is a greater prevalence of sub-clinically elevated impulsivity too.

### Irritability-Aggression

There are multiple lines of evidence for irritability-aggression being associated with low SEP. Crime rates in general are higher in low- than high- SEP communities, but *violent* crime, which is by definition evidence of aggression, shows a greater differential than other crime types (Krivo and Peterson, 1996; Nettle, 2015). The per capita murder rate, for example, is around five times as high in the poorest compared to the most affluent deciles of British neighborhoods (Shaw et al., 2005). There is also evidence for non-linearity in the gradients of violence and homicide: the rates increase continuously with increasing deprivation, but are markedly higher in extremely deprived communities than in moderately deprived ones (Krivo and Peterson, 1996; Shaw et al., 2005).

Several cross-sectional studies of adults have, assessed the triad of anger, hostility and aggression, using psychometric scales. This research has usually been motivated by the known health sequelae of these variables. The studies consistently find some or all components of the triad to be inversely associated with markers of SEP (see Gallo and Matthews, 2003 for a review of 12 such studies). We should also note a remarkable quasi-experimental study in children. Costello et al. (2003) examined the impact on children’s psychopathology of an abrupt increase in family income due to royalties from a casino opened on Native American land. Poor families prior to the royalties had elevated rates of conduct and oppositional/defiant disorder. Irritability and aggression are central to these disorders, and thus the presence of the diagnosed disorders is strong evidence for the presence of the underlying psychological states. In families that began to receive royalties, the prevalence of conduct and oppositional/defiant disorders reduced to the level seen in affluent families, essentially immediately. The prevalence of the disorders did not change in those families that remained poor.

### Anxiety

Several studies have found anxiety disorders to be substantially more common in lower-SEP population groups (Gallo and Matthews, 2003; Stansfeld et al., 2011; Green and Benzeval, 2013). The gradient for anxiety disorders is, in some studies at least, stronger than that for other disorders such as depression (Kessler et al., 1994; Miech et al., 1999). The gradient in sub-clinical experience of anxiety has been less thoroughly studied than that in diagnosed disorder. However, where it has been studied, it has been found, with SEP linearly and inversely related to average experienced anxiety (Warheit et al., 1975; Himmelfarb and Murrell, 1984). The causal nexus in the SEP-anxiety association could point in either direction: low SEP leads to greater anxiety, or greater anxiety leads to loss of SEP. Miech et al. (1999) used prospective longitudinal evidence in young people to attempt to differentiate these two pathways. Since familial SEP predicted anxiety at age 15, anxiety at 15 failed to predict subsequent educational attainment (as a marker of SEP), and educational attainment predicted change in anxiety into adulthood, they concluded that the causal process most consistent with the data was something about low SEP causing anxiety rather than the other way around. A more recent longitudinal study with longer follow up (Stansfeld et al., 2011) suggested that both directions of causality were operative. However, in the natural experiment constituted by the onset of casino royalties, described in the section “Irritability-Aggression,” unlike oppositional/defiant disorder, anxiety symptoms were not reduced by the increase in income (Costello et al., 2003). This was despite poverty being associated with anxiety at baseline in this community.

### Persistent Narcotic Use

The final case study is somewhat different from the first three, in that it concerns a specific behavior rather than broad states. Newly available narcotics are often adopted equally by all social groups, or even preferentially by those of high SEP, who have greater resources to buy them. However, once narcotics become known to be harmful, desistence is much greater in high-SEP groups, so that persistent use becomes more and concentrated in low SEP communities over time. This trajectory has been followed, for example, by tobacco smoking (Lawlor et al., 2003) and cocaine use (Miech, 2008). Much of the concentration of persistent narcotic use in lower-SEP groups is explained by greater likelihood of failure in attempts to quit amongst individuals of lower SEP (Miech, 2008; Hiscock et al., 2012). Their greater likelihood of failure in quitting is attributed, amongst other factors, to their increased impulsivity (see Impulsivity-Hyperactivity).

## Effects of Hunger On Behavior

This section reviews the evidence for the behavioral consequences of hunger, in both humans and in non-human animals. As I will argue, the literature shows that hunger produces a behavioral phenotype centrally characterized by increased impulsivity and hyperactivity, increased irritability and aggression, and increased anxiety, and that has as a side effect greater proneness to use narcotics. Thus, the parallels with the four case studies of SEP gradients discussed in the section “Social Gradients in Behavioral and Psychological Outcomes: Four Case Studies” are clear.

The sources of data on the behavioral consequences of hunger are varied. They include: (1) Evidence from eating disorder patients whose condition involves extreme self-deprivation of food; (2) Evidence from medical food restriction applied as a treatment for morbid obesity; (3) Correlational studies in which behaviors or feelings are associated with hunger or some proxy for it; (4) Evidence from historic semi-starvation experiments with human volunteers; (5) Controlled experimental studies that manipulate moderate hunger in human volunteers; and (6) Studies of non-human animals. Data sources (1)–(5) provide a scale of increasing confidence about the causal consequences of hunger in humans. Sources (1) and (2) involve extreme hunger in highly selected groups whose appetite is potentially disordered in some way in the first place; source (3) is correlational; source (4) is experimental but extreme and involves small, self-selected samples and no control groups; source (5) is fully experimental, incorporates control conditions, and studies variations in hunger within the range of everyday occurrence. Source (6) provides evidence that the human response to hunger is not exceptional from an evolutionary point of view, and at least in part represents a highly conserved pattern. Rather than reviewing the evidence trait by trait as in the previous section, I will briefly describe the relevant findings from each source of data.

### Eating Disorders

Given that eating disorders such as Anorexia Nervosa involve extreme self-control in food ingestion, it seems paradoxical that its most common concomitants include impulsivity, hyperactivity, narcotic use, anxiety and irritability or aggression. Fessler (2002) reviews the evidence for impulsive behaviors in anorexia, describing several observational studies showing that rates of stealing and even armed robbery are surprisingly high during the disorder. Anorexia patients also show elevated impulsivity compared to controls using standard psychometric assessments (Askenazy et al., 1998). They tend to be physically hyperactive (Hebebrand et al., 2003), and eating disorders in general are accompanied by high rates of smoking, alcohol, and drug use (Blinder et al., 2006). Food restriction in eating disorders is strongly associated with anxiety; indeed, *most* inpatients with an eating disorder are diagnosed with a comorbid anxiety disorder (Blinder et al., 2006). Finally, irritability and aggression are very widely reported in eating disorders: in one study, 28% of sufferers experienced uncontrollable anger attacks (Fava et al., 1995); in another, 49% reported having engaged in violence (Thompson et al., 1999). One interpretation of these data is that behavior in eating disorders provides a window onto the psychological consequences of extreme hunger more generally. This is not the only possible interpretation, since this is a group with a severe psychopathology. However, fortunately, there are also other sources of evidence to which we can turn.

### Crash Dieting and Therapeutic Starvation

An historic literature reviewed by Fessler (2002) describes the consequences of crash dieting and ‘therapeutic’ starvation as medical interventions for obese individuals. The consensus of this literature is that the traits of interest all appear as patients become hungrier. Fessler’s (2002, p. 379) summary is worth repeating:

Swanson and Dinello (1970) report that their patients were initially compliant, pleasant, and optimistic, yet became impulsively angry to the point of physical abuse during therapeutic starvation. Swanson and Dinello (1970, p. 124) note “One man asked for help… because he was so angry when in traffic that he feared he would kill any aggravator by smashing his car into them.”

Interestingly, these studies report that the symptoms of impulsivity and aggression seen during treatment constituted marked character reversal, and diminished on refeeding (Fessler, 2002, pp. 378–379). This suggests the symptoms were acute consequences of extreme hunger, not trait-like pre-existing characteristics of the patient group.

### Correlational Studies in General Populations

Correlational studies take a number of different forms. Some are mainly concerned with within-individual comparisons over time: how does the same person feel when he or she is hungry versus satiated? For example, a classic study simply asked participants to describe their sensations and emotions at different stages of hunger and satiety (Monello and Mayer, 1967). Whereas the typical affect during satiety was satisfaction, relaxation, and calmness, hunger was associated with restlessness and excitability. As the hunger became more severe (but still within the everyday range), ‘nervousness’ and irritability became prominent for the majority of respondents. A different type of correlational study makes between-individual comparisons to show that behavioral outcomes of interest are more frequent in individuals in whom hunger occurs more often. For example, Kleinman et al. (1998) used parental interviews to assess the extent to which children in a US sample went hungry (with questions such as ‘Did your child(ren) ever say they were hungry because there was not enough food in the house?’), and also complete a standard measure of psychosocial functioning. Children rated as often hungry had ‘worse’ scores for psychosocial functioning. This is rather a non-specific outcome measure; however, when the researchers analyzed which sub-components of the psychosocial assessment were the ones significantly associated with hunger status, it was two factors called ‘Oppositional behavior/aggression’ and ‘Irritability/anxiety/worry’ (see Murphy et al., 1998b; Slack and Yoo, 2005 for similar findings).

Another source of correlational evidence is the very extensive literature on the psychological correlates of blood glucose levels (see Gailliot and Baumeister, 2007 for review). Blood glucose levels are not synonymous with hunger. They are, however, related: subjective feelings of hunger and desire to eat are associated with brief, transient declines of around 10% in blood glucose levels (Campfield et al., 1996; Melanson et al., 1999), and eating immediately boosts levels of blood glucose. Moreover, experimental infusion of insulin, which artificially induces a decline in blood glucose levels, causes the psychological experience of hunger (Campfield et al., 1996). By implication, participants experiencing low or falling blood glucose levels are either actually hungry, or behaving as if hungry due to problems of glucose regulation. Gailliot and Baumeister (2007) review decades of evidence associating low blood glucose or poor blood glucose regulation with outcomes they encompass under the heading ‘self-control,’ to wit (their terminology): poor attention control; emotion dysregulation; crime, aggression and violence; impulsivity; alcohol use; smoking; and difficulty coping with stress (see also ‘Experimental studies with healthy volunteers’, below).

### Semi-Starvation Experiments

The principal source of experimental semi-starvation evidence is from the Minnesota Semi-Starvation Experiment, in which 36 male volunteers were drastically food-restricted for 24 weeks, and then refed (Keys et al., 1950). The researchers concluded that severe food restriction produced a distinct psychological syndrome that disappeared on refeeding. The symptoms of this syndrome were restlessness, irritability, loss of inhibitions, and poor impulse control. Participants went on impulsive spending sprees, reported violent and angry impulses, and in some cases took risks to the extent of endangering themselves. A rise in accidents and unintended self-injury is also a frequent feature of non-experimental famines (Keys et al., 1950), as is a breakdown of social norms and an increase in social disorder of all kinds (Sorokin, 1942). Minnesota participants increased their consumption of tobacco during the deprivation phase. In summary, the syndrome described in the Minnesota Semi-Starvation Experiment, and by implication also seen in non-experimental famines, resembled an exaggerated form of the low-SEP end of the social gradients described earlier.

### Experimental Studies with General Volunteers

A typical experimental design in this category involves fasting volunteers for a number of hours, followed by either a satiating meal or beverage (satiated condition), or no/non-satiating intake (deprived condition). The outcome is measured standardized behavioral task, and the effect of hunger is estimated using the between-group differences in performance (or change in performance if the measure is repeated). Whereas some studies use a satiating food such as tomato soup (e.g., Kirk and Logue, 1997), those specifically interested in effects of bioavailable glucose use a sugary drink in the experimental condition, and a non-sugary sweet drink in the control condition (Wang and Dvorak, 2010; Aarøe and Petersen, 2013). As explained earlier, hunger, carbohydrate consumption, and blood glucose are all linked, and so for present purposes we will consider all these experiments as informative about the consequences of hunger. Orquin and Kurzban (2016) recently conducted a meta-analysis of the extensive literature in this area (although, note, their set of studies includes both correlational and experimental designs). They found robust evidence across multiple studies that: (1) Hungry participants relative to satiated participants become more willing to work or pay for food, and less willing to work or pay for any kind of non-food reward; (2) Hungry participants relative to satiated have a higher rate of time discounting; that is, they are more impulsive in the waiting sense (interestingly this is true whether or not the reward to be waited for is food-related); and (3) Hungry participants relative to satiated tend to use a more intuitive, snap-judgment decision-making style. There is also experimental evidence for hunger increasing anxiety in human volunteers. In a study by Hausel et al. (2001), surgical patients who were randomly assigned pre-operation to be fasted, to drink water, or to drink a carbohydrate-rich drink. Patients receiving the carbohydrate were significantly less anxious, as well as less hungry, than the other two groups.

### Evidence from Non-human Animals

It is well established that imposing food deprivation in many different species increases locomotor activity (Hebebrand et al., 2003). Intra-specific aggression tends to also increase; this has been documented in taxa as diverse as birds, mammals, and insects (Wallis, 1964; Rohles and Wilson, 1974; Drummond and Garcia Chavelas, 1989). At least part of the increase in aggression is due to the increase in locomotor activity, as active individuals encounter each other more (Wallis, 1964; Rohles and Wilson, 1974). Increased locomotor activity is part of the activation of the motivation to forage. We can think of increased foraging motivation as a kind of disinhibition, since as animals forage more under food deprivation, they expose themselves to greater risk of predation (Godin and Smith, 1988). There is more specific evidence of impulsivity increasing as energetic reserves become lower: Bateson et al. (2015) tested European starlings for relative preference of a smaller-sooner and later-larger food reward, and found that individuals with lower energetic reserves showed a greater preference for the smaller-sooner reward. Finally, there is a mature literature in laboratory animals showing that imposing food deprivation increases consumption of, or preference for, narcotic substances in a dose-dependent manner (see Bell et al., 1997 for review). This has been shown for all major classes of narcotics, including nicotine, cocaine, and alcohol.

### Summary

Multiple lines of evidence converge to suggest that hunger produces a specific behavioral phenotype whose components include impulsivity-hyperactivity, irritability-aggression, anxiety, and a greater propensity to using rewarding narcotics. This evidence is not restricted to extreme food deprivation; hunger within the everyday range has the same consequences in milder form. Indeed, in progressive starvation, anxiety and hyperactivity eventually give way to depression and lethargy; it is mild and moderate hunger that most clearly produces behavioral phenotype described above (Keys et al., 1950). Not only are phenotypic effects of hunger on the four traits of interest established. In some cases, there is evidence of neurobiological mechanisms linking hunger to the behavioral traits too. For example, neuropeptide Y is a brain-expressed neuropeptide whose production is involved in the initiation of eating behavior (Beck, 2006; Nguyen et al., 2012). Elevated expression of neuropeptide Y is also associated with impulsive aggression in both humans and animal models (Coccaro et al., 2012).

Authors on the consequences of hunger have emphasized that the behavioral shifts associated with it do not represent pathology or system failure. Rather, they represent a patterned suite of responses whose function is to prioritize the detection, capture, defense and ingestion of resources in the immediate future over other activities (Loewenstein, 1996; Fessler, 2002; Aarøe and Petersen, 2013; Orquin and Kurzban, 2016). For example, not only does hunger enhance attention to, and increase salience of, food-related stimuli compared to control stimuli (Gilchrist and Nesberg, 1952; Saugstad, 1966); it improves perceptual sensitivity to such stimuli, and hence enhances the ability to detect them rapidly (Radel and Clement-Guillotin, 2012). The suite of responses to hunger is likely to be adaptive, since failure to acquire resources soon when hungry is catastrophic in fitness terms (Higginson et al., 2016). The only one of the four traits under review here that is unlikely to be fitness-enhancing as a response to hunger is increased narcotic use. This is likely to be a maladaptive by-product of the fact that narcotic substances produce a dopaminergic response in mid-brain reward systems that mimics the natural reward signal produced by feeding when hungry (Small et al., 2003).

This and the previous section have laid the groundwork for the argument that several of the documented psychological correlates of having lower SEP are similar to the main known psychological consequences of being hungry. Indeed, the evidence is not just of shared phenotype, but possibly of shared mechanism too. Lower SEP has been shown to correlate with lower dopamine receptor availability in the striatum (Wiers et al., 2016); it is in the striatum that eating pleasant food produces dopamine release (Small et al., 2003). However, the hunger hypothesis as presented in this article is stronger than the claim that low SEP has consequences *like* those of hunger. Rather, it is the claim that some of the sequelae of low SEP are *due to* people of low SEP experiencing more hunger. To establish this claim, it is necessary to demonstrate that people of lower SEP in developed countries do indeed experience a significant burden of hunger. It is to this topic we now turn.

## The Socioeconomic Distribution of Hunger

The claim that people of lower SEP in developed countries might be hungry initially meets with a credibility problem: underweight is very rare in such populations whilst rates of obesity are high, so it seems hard to argue that food is short. Indeed, in developed countries, lower SEP predicts increased probability of obesity, at least for women (Sobal and Stunkard, 1989; McLaren, 2007). However, fat reserves are built up when caloric intake exceeds metabolic requirements averaged over extended periods of time. There is thus no contradiction between overall over-nutrition and having many brief instances of hunger. Indeed, one explanation for overall over-consumption of calories by the poor is as a response to their experience of irregularity in the food supply (Dietz, 1995; Townsend et al., 2001; Nettle et al., 2017).

One way it is possible to put on weight whilst experiencing frequent hunger is by eating less satiating but higher-calorie meals. As we move from higher to lower SEP, diets are composed of progressively less whole grains, vegetables, fruit, and lean meat, and a greater proportion of fats and particularly refined sugars (Drewnowski and Specter, 2004). The consumption of sugar-sweetened beverages, a substantial source of calories in contemporary populations, is strongly socially patterned (Han and Powell, 2013). To a considerable extent, the shift from low- to high-energy density of foods with lower SEP is driven by cost: refined sugars provide many more calories per dollar than fruit or vegetables (Drewnowski and Specter, 2004). However, although energy-dense foods fulfill caloric requirements at low financial cost, they are less satiating than those higher in protein or fiber and lower in sugars (Bornet et al., 2007): that is, hunger returns sooner after eating them.

In addition to lower-SEP meals being less satiating, they may be less regular: studies have found that young people’s omission of breakfast (Hoyland et al., 2012) and of family evening meals (Neumark-Sztainer et al., 2003) is more common in low-SEP groups. It is therefore a reasonable contention that people of lower SEP in developed countries tend to be more often hungry that those of higher SEP, even in the absence of lower total calorie intake or body masses. To demonstrate this unequivocally, one would need to use methods such as experience sampling (Csikszentmihalyi and Larson, 1987) that pinpoint subjective hunger states in time over the course of the participants’ regular lives. Such evidence appears to be lacking at present. However, there is abundant survey evidence based on more global self-reports, to which we now turn.

The prevalence and importance of hunger within populations that are affluent overall began to be appreciated in the United States in the 1980s and 1990s (Kendall et al., 1995; Kleinman et al., 1998). Realizing that a burden of hunger in such populations, if one existed, would not be detectable in body masses, researchers developed two key self-report constructs relating to hunger: food insufficiency (Kleinman et al., 1998), and food insecurity (Kendall et al., 1995; Gundersen et al., 2011).

The concept of food insufficiency was developed particularly in the context of children, and essentially attempts to estimate the incidence of hunger within the life of the child and his or her family. It is assessed using eight questions to parents that specifically probe the occurrence of temporary food shortfall due to resource constraints (i.e., shortfalls due to religious abstinence or other causes are excluded). An example question is “Do your children ever say they are hungry because there is not enough food in the house?”. The responses are most often used to form a discrete classification of children as ‘hungry,’ ‘at risk from hunger’ and ‘not hungry.’ The terms here do not refer to instantaneous states, but the incidence of hunger states over time; they might perhaps be better understood as ‘frequently hungry due to constrained resources,’ ‘occasionally hungry due to constrained resources,’ and ‘never hungry due to constrained resources.’ The social distribution of food insufficiency was extensively studied in the Community Child Hunger Identification Project (CCHIP), a linked series of 18 community studies in different US cities (see Kleinman et al., 1998). The CCHIP showed that food insufficiency was surprisingly prevalent: 8% of children under 12 were classified as ‘hungry’ with another 21% classified as ‘at risk from hunger.’ However, the social distribution was very uneven: in the lowest-income families, the proportion classified as ‘hungry’ rose to 21%, and ‘at risk from hunger’ to 50%. Thus, *most* children from low-income families in the US were classified as either hungry or at risk from hunger.

Food insecurity is defined as the state where the ability to acquire adequate and safe food is limited or uncertain (Kendall et al., 1995). It is routinely assessed in US social and nutritional surveys (Gundersen et al., 2011), and increasingly measured in Latin America and to a lesser extent in other regions too (Nettle et al., 2017). Although food insecurity is not exactly synonymous with hunger, high food insecurity does imply frequent hunger. Indeed, many of the questions in the standard 18-item USDA food insecurity questionnaire (reproduced in Gundersen et al., 2011) in fact address the experience of hunger: for example, “In the last 12 months, did you or other adults in the household ever cut the size of your meals or skip meals because there wasn’t enough money for food?”; and “In the last 12 months, were you ever hungry, but didn’t eat, because you couldn’t afford enough food?”. Again, the responses are used to categorize respondents and their households. Two key categories for our purposes are (the slightly confusingly named) “food insecurity,” which means more than one affirmative response to a food insecurity question, and “very low food security,” which means more than 6 affirmative responses (8 for households with children), and necessarily entails reporting that some household members went hungry at least some times within the last year because of lack of resources. (All households categorized as very low food security by this typology are also food insecure).

The 2008–2009 US prevalence of food insecurity was estimated at around 16%, with around 6% for very low food security (Gundersen et al., 2011). The rate is, however, strongly related to income: of households whose income is half the poverty line, around 40% are classified as food insecure and around 20% as very low food security. This compares to less than 6 and 2% for affluent households. The strong dependence of food insecurity on income is unsurprising, since the construct specifically probes the inability to secure food due to scarce resources. What is important for present purposes is that a large proportion of low-income households report experiencing food insecurity. The implication is that a substantial fraction of people from such households experience an excess of hunger due to their SEP, at least some of the time.

To summarize this section, the available evidence shows clearly that within very affluent populations, individuals of lower SEP eat less satiating diets; do so on more irregular schedules; and a very sizable proportion, at least in the USA, report experiences such as food insufficiency and food insecurity that imply an increased frequency of hunger. Thus, the claim that people of lower SEP are more likely to be hungry at any given time—a necessary assumption of the hunger hypothesis—appears reasonable. However, the hunger hypothesis can be expressed in at least two subtly different versions, each of which makes slightly different predictions. These are the subject of the next section.

## Versions and Testing of the Hunger Hypothesis

The hunger hypothesis posits the following causal relationships: (1) lower SEP leads to more frequent hunger (because it constrains the types of food and the frequencies of high-quality meals that can be procured); and (2) hunger leads to changes in the behavioral and psychological variables of interest (since changes in these variables are part of the adaptive suite of responses to hunger we know exists in humans and other animals). A strength of the hunger hypothesis is that we already know that causal claim (2) is true, since many of the sources of evidence for this arm of the hypothesis (see Effects of Hunger on Behavior) involve experimental manipulation of hunger, in many cases within subjects, thus ruling out alternative causal interpretations.

Given causal claims (1) and (2), the hunger hypothesis predicts that the associations between SEP and the outcome variables of interest will be at least partially statistically mediated by variables to do with hunger. The test of the hypothesis then seems straightforward: measure SEP, measure the behavioral and psychological outcomes, measure hunger, and the rest is multiple regression. However, discussion of testing the hypothesis needs to be qualified by noting that different versions of the hunger hypothesis make slightly different predictions about the nature of the statistical mediation.

### The Current State Version of the Hunger Hypothesis

The boldest version of the hunger hypothesis posits that lower-SEP individuals are on average more impulsive, more irritable, more anxious and more prone to narcotic use when they are studied simply because more of them are hungry at the moment of assessment (or, for outcomes assessed over a longer period, lower SEP participants are more often hungry during the period of observation). This version implies that what have been assumed to be trait-like differences between people of different social positions are in fact not so: they are due to a reversible acute state, one whose occurrence just happens to be non-randomly distributed across social groups. The current-state version of the hypothesis is the most easily falsifiable, since it makes both mediational and interventional predictions. It suggests that in correlational studies, current feelings of hunger should be measured contemporaneously with the other variables, and that current hunger will mediate any associations between SEP and the outcome variable. To spell this out, it predicts that SEP will be associated with current hunger; that current hunger will be associated with the outcome variable; and that any association between SEP and the outcome variable will be substantially attenuated once current hunger is controlled for. It also makes predictions for intervention studies. For example, it suggests that lower-SEP excesses of impulsivity, hyperactivity etc. could be almost immediately reduced by providing regular and satiating meals, whilst high-SEP individuals could be made indistinguishable from those of low SEP by simple food deprivation.

### The Developmental Specialization Version of the Hunger Hypothesis

An alternative version of the hunger hypothesis is the following. Individuals of lower SEP experience acute hunger more often over the course of their lives. This leads to the behavioral propensities and psychological states typical of acute hunger becoming developmentally embedded, to the point where they are expressed (at least to some extent) even when the person is not currently hungry. This would be an example of incremental specialization of behavioral phenotype through multiple developmental exposures (Frankenhuis and Panchanathan, 2011; Panchanathan and Frankenhuis, 2016). The reason for entertaining the developmental specialization version of the hypothesis is the following. A number of recent studies have found that childhood SEP predicts behavior (including behaviors relating to impulsivity and to eating) under certain circumstances, even though current adult SEP does not (Griskevicius et al., 2011a,b, 2013; Mittal and Griskevicius, 2014; Hill et al., 2016). The argument arising from these studies is that childhood experience produces some form of lasting developmental specialization or calibration. Applying the logic of this argument to the hunger hypothesis, we should therefore expect that the individual’s whole developmental experience of hunger, not just their current state of hunger, is relevant to predicting their behavioral phenotype.

Under the developmental specialization version, the overall causal structure of the hypothesis remains the same. However, this version does not make the prediction that *current* hunger will mediate between SEP and the outcome of interest in correlational studies; SEP-outcome relationships should persist regardless of current satiety. Nor does it claim that dietary interventions should immediately abolish any SEP differences. This version is more difficult to falsify, but it is not impossible. For correlational studies, it suggests that what should be developed is some measure of cumulative lifetime exposure to hunger; it is this cumulative measure that should mediate between SEP and the outcome, whilst current hunger should not. Relatedly, it suggests that people with high cumulative exposure to hunger for reasons other than low SEP should look similar in phenotype to those of low SEP. For intervention studies, this version suggests that feeding interventions will not have a dramatic immediate impact, but if continued for a long time, should begin to show effects, effects that will not instantly reverse when the intervention is withdrawn.

The developmental specialization version as I have presented it predicts main effects of cumulative developmental exposure to hunger on behavioral and psychological outcomes. However, hybrid versions of the current state and developmental specialization hypothesis are also possible: developmental exposure to hunger may sensitize individuals to acute exposure. In this scenario, outcomes such as impulsivity and aggression would be predicted by the *interaction* of developmental exposure to hunger and current hunger (Griskevicius et al., 2011a,b, 2013). Neither current hunger without developmental exposure, nor developmental exposure without current hunger, would be sufficient for the phenotype to appear.

We are not currently in a position to adjudicate between the current state and developmental specialization versions of the hunger hypothesis (or a hybrid of the two). They do, however, make critical differential predictions. The current state version of the hypothesis predicts specifically that feeding improvement interventions should greatly attenuate SEP gradients in behavioral and psychological outcomes, and do so rapidly. This exact prediction has not been tested. However, there have been studies of the consequences of breakfast programs for school children. If the current state version of the hypothesis has merit, the effects of such programs on behavioral and psychological outcomes should be large and immediate. There is some evidence that this is the case, with increasing consumption of breakfast associated with immediate psychosocial changes, particularly in rated anxiety and hyperactivity, coupled with improved scholastic performance (Murphy et al., 1998a). It is possible that different outcome variables occupy different positions on the spectrum between immediate state influence and developmental specialization. Of note in this context is the finding in the casino quasi-experiment (Costello et al., 2003) that outcomes to do with aggression responded rapidly to the income boost, while those to do with anxiety did not. [Note, however, that the Costello et al. (2003) results are at variance with those of Murphy et al. (1998a), who detected an immediate change in anxiety in response to the intervention].

### Testability

A key requirement for any testable scientific hypothesis is that it make distinctive predictions. The distinctive central prediction of the hunger hypothesis is that associations between SEP and the outcome variables of interest will be statistically mediated by hunger. However, the sections “The Current State Version of the Hunger Hypothesis” and “The Developmental Specialization Version of the Hunger Hypothesis” have established that this central prediction could in fact be rendered in several ways: a mediating role for current hunger; a mediating role for developmental exposure to hunger; or some kind of interaction between current hunger and developmental exposure. Clearly, this degree of ‘negotiability’ in exactly what the predictions are constitutes something of a theoretical peril. I would therefore recommend that studies initially focus on testing the current state version of the hypothesis, since this is the easiest version to falsify, and the one that is most radical in its implications for policy (see Implications of the Hunger Hypothesis and Recommendations). In the event of the current state version of the hypothesis being falsified, it is still possible that hunger may play a more subtle role in SEP gradients, as specified in a developmental specialization or hybrid version of the hypothesis. That would be a further possibility to turn to in the event of current state of hunger having no explanatory value on its own.

In a sense, we already know that the hungriest people in society are likely to be the most impulsive, most irritable, most anxious and most prone to narcotic use, on average. This is because, from evidence presented above, we know that people of lower SEP tend to exhibit those outcomes (see Social Gradients in Behavioral and Psychological Outcomes: Four Case Studies), and are the most prone to be hungry (see The Socioeconomic Distribution of Hunger). Thus, correlations are very likely to exist between hunger and the outcomes of interest in cross-sectional data. Demonstrating such correlations does not in itself support the hunger hypothesis (which makes causal, not just correlational, claims). Any number of other risk factors for, or consequences of, low SEP could constitute third variables associating hunger to behavioral and psychological outcomes without any direct causal nexus being present. It is for this reason that I have stressed the need to demonstrate statistical mediation of SEP-behavior associations by hunger. Many possible alternative explanations would be compatible with SEP, hunger, and behavioral outcomes all being related to one another. Mostly, they would not predict that the inclusion of the indirect pathway via hunger would substantially attenuate the direct relationship between SEP and the outcome variable. The mediation prediction generally implies that the associations between SEP and hunger, and between hunger and the outcome, will be stronger than the simple association between SEP and the outcome.

Demonstrating statistical mediation, though, is still a relatively weak form of causal inference. For one thing, it does not necessarily exclude reverse causality (see Alternative Pathways). Stronger evidence for the causal account specified by the hunger hypothesis would come from experimental and quasi-experimental designs in which hunger or an instrument closely related to it were randomly or quasi-randomly varied. I have already mentioned feeding improvement interventions such as breakfast programs, which provide a stronger test of the hypothesis. Randomized control trials of such interventions—not just for children, and not just for scholastic outcomes—are a research priority. In addition, food assistance programs, which vary from jurisdiction to jurisdiction and can have a substantial impact on hunger (Ratcliffe et al., 2011) may provide quasi-experimental opportunities, as they have in the study of the association between food insecurity and obesity (DeBono et al., 2012).

### Alternative Pathways

The purpose of this article is to lay out the grounds for further investigating the hunger hypothesis, not to claim that it is true. The critical studies have not yet been conducted. Nonetheless, it is clear even from what is known so far that there are alternative pathways that may contribute to the three-way associations of SEP, hunger, and behavioral outcomes. Some care will be required attempting to dissect out the contributions of these different pathways.

First, as mentioned above, there is a reasonable amount of evidence for the operation of reverse causality in associations between SEP and anxiety, and SEP and impulsivity. That is, in several longitudinal studies, experiencing anxiety problems (Stansfeld et al., 2011), or exhibiting impulsivity (Moffitt et al., 2011), predicts later downward social mobility and hence lower final SEP. This is inconsistent with the causal structure discussed so far: it implies that early-life anxiety- or impulsivity-proneness leads to lower SEP, which leads to more hunger. There are two points to make about reverse causality. The first is that the two directions are not mutually exclusive; both direct and reverse causality can operate over the life course (Stansfeld et al., 2011), constituting a feedback process. The second is that, given we already know that hunger has a causal impact on anxiety and impulsivity (see Effects of Hunger on Behavior), we should expect, even if reverse causality has an important role in the SEP-anxiety and SEP-impulsivity associations, that interventions or policy changes that reduce hunger would still ease the burden of anxiety and impulsivity in low SEP groups. In fact, such interventions might be particularly valuable in such groups if those groups contain a relatively high proportion of people temperamentally vulnerable to anxiety problems (Tambs et al., 2012).

Second, traditions of research on aggregate outcomes (e.g., variation in average levels of anxiety, or of violence, across countries) have implicated causal factors that are not themselves hunger, but may be associated with hunger. For example, anxiety has been found to be particularly frequent or elevated in rural Zimbabwe (Langhaug et al., 2010), the Central African Republic (Vinck, 2017), and the Gaza strip in the wake of the second uprising (Elbedour et al., 2007). These findings could be interpreted through the lens of the hunger hypothesis, since all of these places are likely to feature a substantial hunger burden, but the findings could equally well be interpreted in terms of levels of ‘threat’ more generally (including the threat of violence, which may or may not in turn be related to hunger). Comparative research on interpersonal violence has found it to be predicted by national poverty, and being located in the tropics (Burke et al., 2015; van Lange et al., 2017). Again, this could relate to the burden of hunger, but contemporary theorizing has focussed more on direct behavioral consequences of climate factors such as high temperatures. Given that adverse ecological factors (hunger, socio-political instability, and extreme climate) tend to co-occur, dissecting out the contributions of different causal pathways is extremely challenging. This is especially true with aggregate data, but applies to individual-level data too. This reinforces the conclusion of the previous section that experimental, quasi-experimental, and instrumental-variable study designs are a key priority.

As a final puzzle, there is cross-national variation in SEP gradients in outcomes such as anxiety. If hunger were the main operative factor, one might expect that SEP gradients in anxiety would be more marked in countries where the poor are very poor (hence hungry), and much less marked in countries such as Norway which are relatively economically equal with strong social protection. However, this does not appear to be the case: the gradient in anxiety disorder in Norway is steeper than many that have been observed in developing countries (Tambs et al., 2012). In Japan, it appears the gradient may even be reversed, at least for childhood SEP (Ochi et al., 2014). There are a number of possible explanations for these findings, not least involving sociocultural differences in diagnosis and reporting of medical disorders. Nonetheless, it remains to be seen whether and how these variations could be reconciled with the hunger hypothesis.

## Implications of the Hunger Hypothesis and Recommendations

I conclude with some implications of the hunger hypothesis (discussed below), and some recommendations for research (**Table 1**). The first of the implications is that the hunger hypothesis implies a more direct link between economic factors and psychological outcomes than is usual. To a considerable extent, food insufficiency and food insecurity are driven by available money; after all, their measures explicitly ask about the inability to procure healthy, balanced meals three times a day due to lack of financial resources, not for any other reasons. Available budget to spend is also a very good predictor of diet quality (Drewnowski and Specter, 2004). So if the hunger hypothesis is correct, then disposable income should be a direct driver of rates of impulsivity, anxiety, aggression, and substance abuse. There is some evidence consistent with this view: for example, financial difficulties in the family are the strongest single predictor of child ADHD (Russell et al., 2015a); and conduct and oppositional-defiant disorder (though not anxiety) were radically reduced in a natural experiment that increased family income but left other factors such as education unchanged (Costello et al., 2003). This has strong implications for the evaluation of policy alternatives likely to change the distribution of income across social groups, and the likely impact of changing economic conditions. For example, the crisis of 2008–2009 produced a sharp increase in the prevalence food insecurity in US families (Gundersen et al., 2011). Under the hunger hypothesis, this should also have produced increases in impulsivity, anxiety and aggression, though the predicted pattern and timing of these increases would depend on the version of the hypothesis—current state, developmental specialization or hybrid—is adopted.

**Table 1:** Recommendations for research arising from the hunger hypothesis.

1	Experience sampling should be used to map the experience of hunger over time during normal life for different social groups
2	Hunger (or where appropriate a related variable such as food insecurity) should always be measured in studies of SEP and behavioral or psychological outcomes, and considered as a key mediating variable
3	Experimental and intervention studies should investigate whether feeding can attenuate or abolish SEP differentials in behavior, not just in children but also in adults
4	Effects of policy changes that alter incomes or food security should be studied broadly to include not just physical health but behavioral and psychological outcomes
5	Measuring, and understanding the psychological consequences of, cumulative experience of hunger over the life course should be research priorities
6	The hypothesis that individuals of lower SEP show the cognitive profile of acutely hungry people, including both processing costs and processing advantages of that state, should be investigated

A second implication of the hunger hypothesis concerns the malleability of SEP differences in behavioral and psychological outcomes. These differences are often conceptualized as ingrained and difficult to change. The hunger hypothesis instead lays them at the door of a reversible acute state, with the strong implication that they could therefore be rapidly eliminated. This is a style of explanation (enduring patterns of individual differences explained in terms of transient visceral states) not often encountered; the only other example with which I am familiar is Van Cauter and Spiegel’s (1999) parallel argument about the role of sleep in the relationship between SEP and health. The extent of the proposed malleability varies among the versions of the hypothesis: the current state version sees them as radically malleable and therefore reversible essentially instantaneously. The developmental specialization version sees them as malleable over the rather slower timescale of the life course. All versions agree that any social interventions or policy measures that alleviate food insufficiency and insecurity will have widespread effects that extend well beyond narrowly nutritional outcomes. Such interventions and measures should therefore, according to the hunger hypothesis, be accorded special consideration.

A third implication arises from the idea that the suite of traits associated with hunger is not a system failure, but an adaptation. That is, the psychological shifts brought on by hunger exist because (on average, over evolutionary time) they improve the location, capture and defense of food resources. In other words, these traits make individuals *better than they would otherwise be* at achieving a certain class of goals, albeit at the expenses of other classes of goals. If we accept that the psychological phenotype associated with low SEP stems from an adaptive response to hunger, then we should predict that individuals of lower SEP will be relatively advantaged at certain types of task, even if they are relatively disadvantaged at others. Research into social gradients in behavior is so deeply imbued with assumptions of deficit that these possibilities have been little explored (see Frankenhuis and de Weerth, 2013 for a related discussion). The hunger hypothesis suggests ways this possibility might be opened up. For example, children deprived of breakfast show a less focussed attention style, leading to poorer performance on many tasks. However, in one study they actually showed an advantage in incidental learning (recall of sources of information they had not been asked to attend to) (Pollitt et al., 1982). The hunger hypothesis predicts this will be true of low-SEP children more generally. It also predicts that low-SEP individuals would show a relative advantage on tasks broadly isomorphic to foraging or resource defense, for example involving rapid detection of food-related stimuli (Radel and Clement-Guillotin, 2012).

## Conclusion

I have here presented the hypothesis that hunger could play an important role in some social gradients in behavioral and psychological outcomes within affluent countries. It is possible to establish: (a) that a suite of variables including impulsivity-hyperactivity, persistent narcotic use, anxiety, and irritability-aggression is consistently associated with measures of low SEP; (b) that hunger increases these same four variables; and (c) that there is a burden of hunger within affluent societies, at least the USA, and it falls especially on those of lower SEP. I cannot claim to have shown that (b) and (c) add up to an explanation of (a). However, the idea that they might provide at least a partial explanation is reasonable and deserves further investigation. In addition, as I have stressed, there are subtly different versions of the hypothesis that give slightly different accounts of *how* facts (b) and (c) contribute to fact (a), accounts that lead to subtly different predictions. The hunger hypothesis certainly provides a novel and provocative view of a complex set of issues concerning how social inequalities (in this case, in the distribution of financial and nutritional resources) relate to variation in individual behavior.

## Author Contributions

The author confirms being the sole contributor of this work and approved it for publication.

## Conflict of Interest Statement

The author declares that the research was conducted in the absence of any commercial or financial relationships that could be construed as a potential conflict of interest.
